# TIPE2 Protects Bronchial Epithelial Cells From Pyroptosis and Oxidative Stress by Interacting With the E3 Ubiquitin Ligase FBW1A in Asthmatic Mice

**DOI:** 10.1002/jbt.70534

**Published:** 2025-10-10

**Authors:** Wei Li, Yonghong Zhang, Yuanyuan Wu, Dan Wang, Juan Ge, Hongyan Zhao, Yun Liu

**Affiliations:** ^1^ Department of Respiratory and Critical Care Medicine Second Affiliated Hospital of Xi'an JiaoTong University Xi'an Xincheng District China

**Keywords:** asthmatic bronchial epithelial cells, C1qbp associated mitochondrial damage and oxidative stress, NLRP3 associated pyroptosis, the ubiquitinase FBW1A, TIPE2

## Abstract

Dysfunction of respiratory epithelial cells is acknowledged as an important pathogenesis of asthma. Tumor necrosis factor‐α induced protein 8 like‐2 (TNFAIP8L2 or TIPE2) is a famous suppressor of hyperinflammation, which has been involved in regulating asthma progression, however, its role in pyroptosis and oxidative stress of bronchial epithelial cells is still unclear.

1 μg/mL LPS and 50 ng/mL IL‐13 was used to induce dysfunction of human bronchial epithelial cells BEAS‐2B, and TIPE2 expression was detected at different time points after incubation. Next, TIPE2 expression vector (pcDNA‐TIPE2) was transfected into BEAS‐2B cells to investigate its involvement in LPS/IL‐13‐induced pyroptosis and oxidative stress. Then, bioinformatics analysis and co‐immunoprecipitation(co‐IP) assay were used to verify the binding protein of TIPE2, and rescue experiments were performed to validate the molecular mechanism through which TIPE2 regulated pyroptosis and oxidative stress of BEAS‐2B cells. Finally, ovalbumin (OVA) was used to establish an asthmatic animal model in Balb/c female mice, adenovirus‐mediated overexpression vector of TIPE2 (Ad‐TIPE2) was applied to administrate the asthmatic mice, and the effect of TIPE2 on the progression of asthma was investigated. LPS/IL‐13 treatment induced cell death (PI^+^ cells) and TIPE2 downregulation in a time‐dependent manner. LPS/IL‐13 treatment promoted NLRP3‐mediated pyroptosis, secretion of pro‐inflammatory cytokines, and oxidative stress in BEAS‐2B cells, while overexpression of TIPE2 was able to largely reverse the effect of LPS/IL‐13. TIPE2 directly interacted with the E3 ubiquitin ligase β‐Transducin Repeat‐containing protein (FBW1A) and positively regulated its expression. Rescue experiments with recombinant ubiquitin protein (Ub) and the ubiquitination inhibitor MG132 demonstrated that F‐box and WD repeat protein 1A (FBW1A)‐mediated ubiquitination of NLRP3 and C1qbp is required in TIPE2 deficiency‐induced pyroptosis, mitochondrial damage and oxidative stress. Overexpression of TIPE2 suppressed NLRP3‐mediated pyroptosis and C1qbp‐mediated oxidative stress and improved respiratory function and pulmonary tissue damage in vivo. TIPE2 protects asthmatic bronchial epithelial cells from NLRP3‐induced pyroptosis and C1qbp‐induced oxidative stress by interacting with the E3 ubiquitin ligase FBW1A in vitro and in vivo.

## Graphical abstract

TIPE2 directly interacted with the E3 ubiquitin ligase FBW1A and positively regulated its expression. TIPE2/FBW1A complex could regulate ubiquitination degradation of C1qbp and then inhibit NLRP3 inflammasome activation to suppress oxidative stress and inflammation in bronchial epithelial cells, thus improved respiratory function and pulmonary tissue damage in asthma.

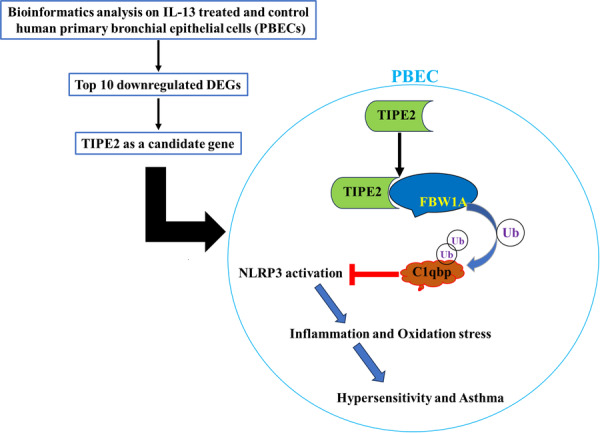

**Abbreviations**
anti‐Drp1: Anti‐dynamin‐related protein 1
anti‐Mfn2: Anti‐Mitofusin 2
ASC: apoptosis‐associated speck‐like protein containing a caspase activating recruitment domain
C1qbp: complement 1q‐binding protein
FBW1A: β‐Transducin Repeat‐containing protein
MFN2: mitochondrial fusion marker protein mitofusin 2
NLRP3: NOD‐like receptor protein 3
TNFAIP8L2(TIPE2): Tumor necrosis factor‐α induced protein 8 like‐2

## 1 Introduction

Asthma is a common chronic respiratory disease, with high heterogeneity and characterized by chronic airway inflammation and airway hyperresponsiveness. Currently, asthma affects more than 350 million people worldwide, and more than 20 million severe patients are facing the threat of death. Moreover, the incidence of asthma is increasing, and the age of onset tends to be younger [1]. However, the treatment effect of asthma in clinical is still far away from ideal, especially for adult and severe asthma patients. Therefore, it is urgent to deeply understand the pathogenesis of asthma and discover reliable therapeutic targets.

The main events during asthma occurrence and development include excessive airway epithelium damage and bronchoconstriction, infiltration and aggregation of immune cells, mucus hypersecretion, inflammation responses, and oxidative stress. The respiratory epithelial cells supply a physical, functional, and immunologic protective screen for the respiratory tissues against the harming factors, and dysfunction of respiratory epithelial cells is acknowledged as an important pathogenesis of asthma, such as abnormal metabolism and secretion, death, stress, and increased permeability [2]. Numerous studies have demonstrated that dysfunction of bronchial or alveolar epithelial cells is frequently observed in asthma and may be a major driver of asthma progression. For example, fibroblast growth factor 2 (FGF2) was upregulated in bronchial and alveolar epithelium of asthma patients and asthmatic mouse lung, which aggravated airway inflammatory cell infiltration and promoted release levels of pro‐inflammatory cytokines via the FGFR/mitogen‐activated protein kinase (MAPK)/nuclear factor kappa B (NF‐κB) pathway [3]. Studies in ovalbumin (OVA)‐induced asthmatic mice and LPS/IL‐13‐induced cellular asthmatic model in bronchial epithelial cells revealed that secretion of pro‐inflammatory cytokines and level of oxidative stress were robust in asthma, and repression of inflammation and oxidative stress was an effective measurement to alleviate asthma [4, 5].

Pyroptosis is a caspase‐1 or caspase‐4/5/11‐dependent programmed cell death associated with significant inflammation, which is initiated by inflammasomes or cytosolic LPS in innate immunity. When caspase‐1 is activated, Gasdermin‐D rapidly forms pores in the plasma membrane, allowing for osmotic lysis and release of inflammatory cytokines and cell contents, in contrast to apoptosis. Both types of pyroptosis lead to the release of potent inducers of inflammasome activation and consequent inflammation [6]. The inflammasome is a multiprotein complex, and it can be divided into subtypes based on the different combinations of molecules [7]. The most well‐known inflammasome is the NOD‐like receptor thermal protein domain associated protein 3 (NLRP3) inflammasome, which is mainly composed of NLRP3, apoptosis‐associated speck‐like protein containing a caspase activating recruitment domain (ASC) and caspase‐1, which are assembled to react to microbial infections or endogenous danger signals and function as a key sensor in the pyroptosis signaling pathway and governed the secretion of interleukin‐1β (IL‐1β) and the inflammatory response [8]. The activation of NLRP3 further induces pyroptosis by recruiting ASC and activating caspase‐1 to shear pro‐IL‐1β and release mature IL‐1β, which orchestrated infection and immune response to cellular stress, which contributes importantly to many major diseases, including asthma [9, 10].

Oxidative stress is also a key pathophysiological component of respiratory diseases, including asthma. Oxidative stress refers to an imbalance of oxidation–reduction response, leading to excessive production of free radicals and other oxidative substances, thereby causing damage to cells and tissues [11]. As acknowledged, the imbalance between oxidative stress and antioxidant defense serves as a fundamental factor of inflammation in asthma. Evidence suggests that prenatal exposure to sources of reactive oxygen species (ROS), such as tobacco smoke, polluted air, and reactive dyes, is associated with asthma [12]. Other postnatal factors that can induce oxidative stress also contribute importantly to asthma, such as infections, obesity‐induced lipid peroxidation, and lack of biological antioxidants [13]. Moreover, oxidative stress was reported to be related to uncontrolled asthma in asthma patients, and oxidative stress‐related kinases, transcription factors or receptors, such as nuclear factor kappa B (NF‐κB), NF‐E2‐related factor 2 (NRF2), Toll‐like‐receptors (TLRs) and complement 1q‐binding protein (C1qbp), have been regarded as potential therapeutic targets for asthma [14, 15, 16, 17].

In this study, we re‐analyzed the data set GSE206510 from the GEO (Gene Expression Omnibus) online database and searched for potential key regulators in the dysfunction of bronchial epithelial cells, which revealed that TIPE2, also TNFAIP8L2, was the most downregulated gene in IL‐13‐treated bronchial epithelial cells. Then, we investigated the protective role of TIPE2 in LPS/IL‐13‐incubated bronchial epithelial cell pyroptosis and oxidative stress and the underlying mechanism in vitro and in vivo.

## 2 Materials and Methods

### 2.1 Reagents

Lipopolysaccharides (LPS, derived from *Escherichia coli* O127: B8), Interleukin‐13 (IL‐13) were purchased from Sigma‐Aldrich, St. Louis, MO. The total RNA isolation system, reverse transcription system and SYBR Green PCR Master Mix were purchased from TIANGEN BIOTECH, Co. Ltd. Beijing, China. The anti‐TIPE2 antibody was purchased from Proteintech Inc. Rosemount, MN. Anti‐TIPE2, anti‐FBW1A, anti‐C1qbp, anti‐NLRP3, anti‐Drp1, anti‐Mitofusin 2 (anti‐Mfn2), anti‐ASC, anti‐cleaved‐caspase 1, anti‐Gasdermin D, and anti‐cleaved‐Gasdermin D antibodies were purchased from Abcam, Cambridge, UK. The ELISA kit of IL‐1β, IL‐6, TNF‐α, IFN‐γ, MCP‐1, and IL‐12 was purchased from Dakewei, Shanghai, China. ROS fluorescence probe (DCFH‐DA), MDA (malondialdehyde) Detection Kit and PI (Propidium Iodide) dyeing solution, and DAPI (diamidino‐2‐phenylindole) dyeing solution were purchased from Sigma‐Aldrich.

### 2.2 Cell Culture

Human bronchial epithelial cells BEAS‐2B were purchased from the Southern Cell Company (Guangzhou, China). Cells were cultured in Dulbecco's modified Eagle medium (DMEM) containing 10% fetal bovine serum (FBS, Hyclone) and 1% penicillin‑streptomycin at 37°C in a humidified 5% CO_2_ atmosphere. Next, we put bronchial epithelial cells into a sterile 96‐well plate and incubated with 1 μg/mL LPS and 50 ng/mL for indicated times.

### 2.3 Cell Transfection

Small interference RNA to TIPE2 (si‐TIPE2) or overexpression RNA to TIPE2 (pcDNA‐TIPE2) was synthesized by Genchem Co. Shanghai, China. The DNA target sequence of TIPE2 was 5′‐GAA GTG AAA CTC AGG TCC G‐3′, and the scrambled control RNA was 5′‐TTC TCC GAA CGT GTC ACG T‐3′. Cells were infected with recombinant lentiviruses carrying si‐TIPE2 or pcDNA‐TIPE2 according to the manufacture's instruction and infected with the enhanced infectious solution(Lipofectamine 3000, Invitrogen). The transfection efficiency of bronchial epithelial cells transfected with lentiviral vectors was observed by fluorescence microscopy at 3 days. The results showed that the transfection efficiency was > 90% at a multiplicity of infection (MOI) = 10.

### 2.4 CCK‐8 Assay

Bronchial epithelial cells were grown to sub‐confluence and seeded in triplicate wells of 96‐well plates at a concentration of 1 × 104 cells/well in a final volume of 200 μL and allowed to incubate overnight at 37°C. Then, 10 μL of CCK‐8 solution was added into each well, and the plates were incubated for 2 h at the same incubator conditions. The graph was prepared according to the absorbance value, which was read at 450 nm.

### 2.5 PI Staining

Bronchial epithelial cell death was assessed with PI, which can stain the nuclei of cells with loss of the integrity of the plasma membrane. Bronchial epithelial cells were rinsed twice with PBS, and then they were incubated with PI dye solution (10 μg/mL) for 15 min at 37°C and subsequently fixed with 4% paraformaldehyde for 15 min. Then, Bronchial epithelial cells were counterstained with DAPI for 2 min and visualized under a fluorescence microscope (Leica DMI 4000B, Wetzlar, Germany). Twenty high‐power fields were randomly chosen and blindly quantitated. The number of PI + /DAPI+ positive cells (necrotic cells) was expressed as a percent of the total cells.

### 2.6 RT‐qPCR Assay

Cells were collected at the indicated time points, and total RNA was extracted using a single extraction with an acid guanidinium thiocyanate‐phenol‐chloroform mixture according to the manufacturer's instructions (Promega, Madison, WI). The concentration of purified total RNA was determined spectrophotometrically at 260 nm. After the removal of potentially contaminating DNA with DNase I, 2 μg of total RNA from each sample was used for reverse transcription with oligo dT primers and SuperScript II to generate first‐strand cDNA. TIPE2 mRNA expression was quantified in duplicate by two‐step real‐time RT‐qPCR with SYBR Green. As the internal control, GAPDH was analyzed under the same conditions using the appropriate primers. The PCR mixture was prepared by using SYBR Green PCR Master Mix with the primer for TIPE2 were forward 5′‐GAA GTG AAA CTC AGG TCC G‐3′ and reverse 5′‐TTC TCC GAA CGT GTC ACG T‐3′. The thermal cycling conditions were 15 min at 95°C, followed by 40 cycles of 95°C for 10 s and 60°C for 32 s in a sequence detection system. GAPDH was used as a housekeeping gene for normalization, and the relative changes in gene expression were analyzed by the 2^−ΔΔCT^ method.

### 2.7 Western Blot Analysis Assay

Lung tissues or BEAS‐2B cells were collected and washed with cold PBS and lysed at a density of 1 × 10^7^ cells/100 μL of lysis buffer (150 mM NaCl, 1.0% NP‐40 or 0.1% Triton X‐100, 0.5% sodium deoxycholate, 0.1% SDS, 50 mM Tris‐HCl (pH 8.0), and protease inhibitors). After incubation on ice for 30 min, the homogenate was centrifuged at 14,000 rpm for 30 min at 4°C, and the protein contents in the supernatant were measured with a Bradford protein assay kit. Thirty micrograms of total protein from the supernatants were boiled at 95°C for 5 min after mixing with loading buffer at a 4:1 ratio, separated by 12% SDS‐PAGE, and transferred to polyvinylidene fluoride (PVDF) membranes. Specific antibodies were used. An anti‐β‐actin or anti‐COXIV rabbit polyclonal antibody (Applygen Technologies Inc. Beijing, China) was used as a loading control. All immunoblots were visualized by enhanced chemiluminescence (ECL) reagents. The protein levels were quantified by densitometry (Bio‐Rad Laboratories, Hercules, CA).

### 2.8 Enzyme‐Linked Immunosorbent Assay (ELISA)

The activity levels of LDH and caspase‐1 were detected by using the ELISA kits (Roche, Basel, Switzerland) according to the manufacturer's instructions. The expression of cytokines, including IL‐1β, IL‐6, IL‐18, tumor necrosis factor‐α (TNF‐α), and monocyte chemotactic protein 1 (MCP‐1) as well as interferon‐γ (IFN‐γ), was detected by commercially available enzyme‐linked immunosorbent assay (ELISA) kits, and all the procedures were strictly according to the instructions of the manufacturer (Dakewei, Shanghai, China).

### 2.9 Detection of MDA and ROS Contents

Malondialdehyde (MDA) and reactive oxygen species (ROS) contents in the cells were detected with the MDA Detection Kit and the ROS fluorescence probe (DCFH‐DA), respectively, according to the instructions of the manufacturer.

### 2.10 Co‐Immunoprecipitation (Co‐IP) Assay

BEAS‐2B cells were lysed using the Co‐immunoprecipitation (IP) buffer (120 mM NaCl, 1 mM EDTA, 40 mM HEPES, pH 7.4, 50 mM NaF, 10 mM beta‐glycerophosphate, 0.3% CHAPS, 1 mM Na_3_VO_4_, 1 mM PMSF, 10 mg/ml leupeptin,10 mg/ml aprotinin, and 10 mM MgCl_2_). Lysates were centrifuged at 12000 × g for 30 min at 4°C and incubated with primary antibody for 1 h. Protein A ‐Sepharose (Santa Cruz Biotechnology, sc‐2003) was added and incubated overnight at 4°C with rotation. Then the protein complexes were washed 5 times with Co‐IP buffer and boiled for 5 min, followed by SDS‐PAGE and western blot analysis assay.

### 2.11 Establishment of Asthmatic Mouse Model and TIPE2 Overexpression In Vivo

Female Balb/c mice (8 weeks, weighing 17–19 g) were kept in the Experimental Animal Center of Xi'an Jiaotong University (Xi'an, China). For establishment of the asthmatic model, 0.2% OVA (dissolved in 4% aluminum hydroxide gel) were injected into mice through the eight‐point method (25 μL per point on both hind legs, 25 μL mL per point on both groins, 50 μL per point on the back, 100 μL in the neck, and 200 μL in the abdominal cavity respectively) at day 0 and day 14, and, at day 22, 1% OVA (ovalbumin, dissolved in physiological saline solution) was applied in atomization administration of the injected mice 45 min/day for 7 consecutive days. Mice injected with equivalent 4% aluminum hydroxide gel without OVA were regarded as the control. For TIPE2 overexpression in vivo, 200 μL (10^12^ PFU/mL) of adenoviral TIPE2 overexpression vector (TIPE2) and the negative control vector (Vector), constructed and packaged by Ribobio Biotechnology Co. Ltd. (Guangzhou, China) were administrated into the model mice via tail vein injection at day 14 to investigate the effect of TIPE2 overexpression on asthma progression in vivo. At day 29 post the first injection, mice in each group were applied in the assays for lung function, cell counts in bronchoalveolar lavage fluid, lung tissue morphology, and expression of inflammatory factors and related regulatory genes.

### 2.12 Statistical Analysis

All data are presented as mean ± SEM. Statistical analysis was performed by using SPSS Statistics version 20.0 software. Multigroup comparisons of the means were evaluated by using one‐way analysis of variance (ANOVA) test, and Student's *t*‐test was performed for two‐group comparisons. The significance level was set at *p* < 0.05.

## 3 Results

### 3.1 TIPE2 Was Screened as a Potential Regulator in Asthma

**Figure 1:**
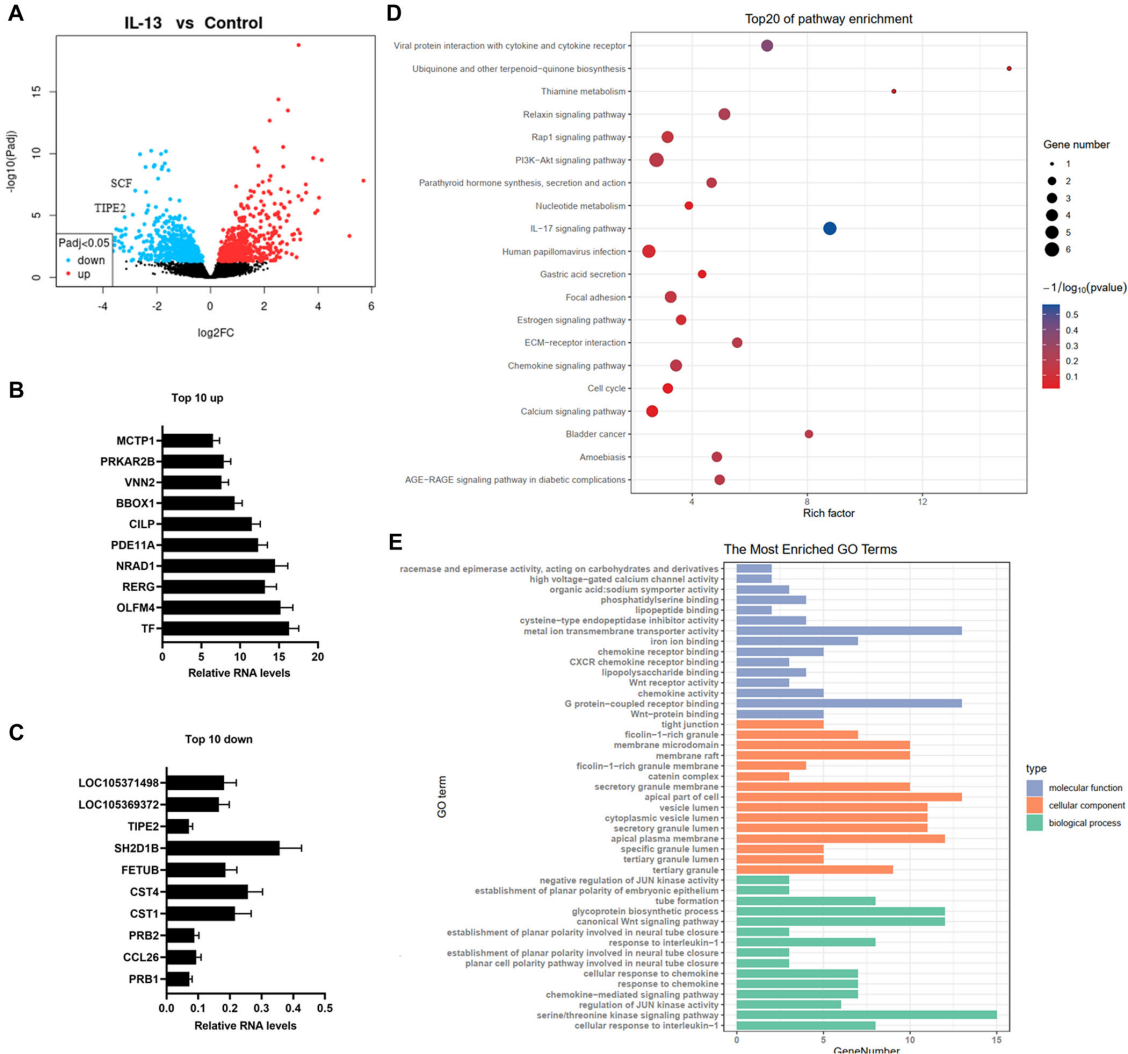
TIPE2 was screened as a key regulator in asthmatic bronchial epithelial cells. The data set GSE206510 was downloaded from the GEO database. We re‐analyzed the data of GSE206510 and obtained potential key regulators in the dysfunction of bronchial epithelial cells. (A) Volcano plots were used to show the differentially expressed genes (DEGs). Then, qPCR analysis was used to validate the top 10 upregulated (B) and top 10 downregulated (C) genes in the sequencing results. (D, E). KEGG and GO analyses were used to annotate the DEGs.

The data set GSE206510, which includes the RNA sequencing data of primary human bronchial epithelial cells derived from control and asthmatic patients, stimulated with IL‐13, was downloaded from the GEO database. We re‐analyzed the data of GSE206510 and obtained potential key regulators in the dysfunction of bronchial epithelial cells. Differentially expressed genes (DEGs) were shown in Figure 1A, which included 581 upregulated and 536 downregulated genes. Then, qPCR analysis was used to validated the top 10 upregulated and top 10 downregulated genes in the sequencing results, and the results showed that the qPCR results were basically consistent to the sequencing results (Figure 1B and C), whereas, it is worth mentioning that TIPE2, ranking 8th of the most downregulated genes, was the most downregulated gene in the qPCR analysis. KEGG and GO analyses revealed that these DEGs are enriched in multiple kinase, infection, and ion metabolism pathways, such as PI3K‐AKT, papillomavirus infection and calcium signaling pathways, and associated with chemokine activity and responses (Figure 1D and E).

### 3.2 TIPE2 Was Downregulated in LPS/IL‐13‐Induced Bronchial Epithelial Cells

**Figure 2:**
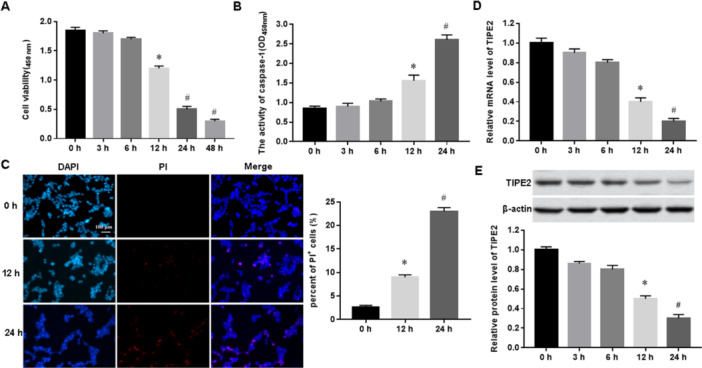
TIPE2 was downregulated in LPS/IL‐13‐induced bronchial epithelial cells. Bronchial epithelial cells were incubated with 1 μg/mL LPS for 5 h, then incubated with 5.0 mM IL‐13 for 1 h, cultured at 37°C for 48 h, and cells were collected at 0, 3, 6, 12, 24 and 48 h, respectively. (A) CCK‐8 assay was performed to detect cell viability. (B) The activity of caspase‐1 was measured with ELISA. (C) PI staining was used to detect the number of pyroptosis. (D) The mRNA expression of TIPE2 was detected by using RT‐qPCR. (E) The protein levels of TIPE2 were measured with Western blot analysis. *N* = 5, compared with 0 h, *p < 0.05; compared with 0 h and 12 h, ^#^ *p* < 0.05.

Then, to investigate the function of TIPE2 in asthma in vitro, 1 μg/mL LPS and 50 ng/mL IL‐13 was used to incubate human bronchial epithelial cells BEAS‐2B. Our results showed that the viability of bronchial epithelial cells (Figure 2A) began to decrease after 12 h of LPS/IL‐13 treatment and significantly decreased after 24 h of treatment. Next, we examined the number of PI‐positive cells and caspase‐1 activity after 0 h, 12 h, and 24 h of LPS/IL‐13 treatment, respectively, and found that caspase‐1 activity (Figure 2B) and the number of PI‐positive cells (Figure 1C) was increased in a time‐dependent with LPS/IL‐13 treatment. These results indicated that we successfully constructed a pyroptosis model of bronchial epithelial cells by LPS/IL‐13 induction. In addition, we also found a time‐dependent decrease in TIPE2 mRNA (Figure 2D) and protein (Figure 2E) expression in LPS/IL‐13‐induced bronchial epithelial cells.

### 3.3 Overexpression of TIPE2 Inhibits Bronchial Epithelial Cell Pyroptosis Induced by LPS/IL‐13

**Figure 3:**
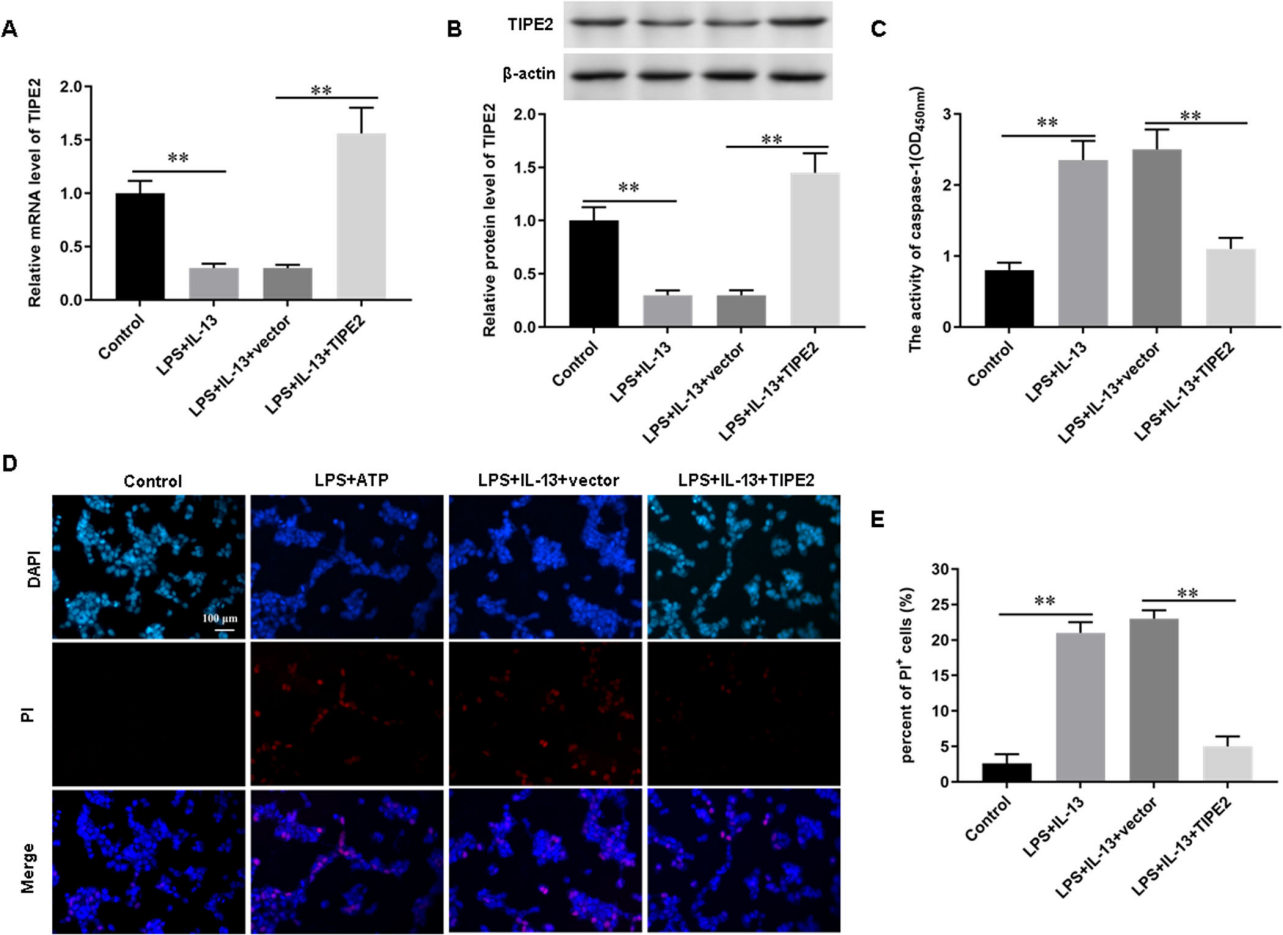
Overexpression of TIPE2 inhibits pyroptosis of bronchial epithelial cells. TIPE2 expression vector (pcDNA‐TIPE2) or pcDNA‐empty vector was transfected into bronchial epithelial cells, transfected for 48 h, and then cells were collected. (A) The mRNA level of TIPE2 was detected by using RT‐qPCR. (B) The protein levels of TIPE2 were detected by using Western blot analysis. (C) The activity of caspase‐1 was measured with ELISA. (D, E) PI staining was used to detect the number of pyroptosis. *N* = 5, *p < 0.05, **p < 0.01.

To confirm whether TIPE2 is involved in the dysfunction of bronchial epithelial cells, we transfected bronchial epithelial cells with a TIPE2 overexpression vector (pcDNA‐TIPE2) and found that TIPE2 mRNA (Figure 3A) and protein (Figure 3B) expression levels were significantly upregulated in pcDNA‐TIPE2‐transfected bronchial epithelial cells. In addition, we also found that the activity level of caspase‐1 (Figure 3C) in LPS/IL‐13‐induced bronchial epithelial cells was significantly upregulated, and the number of PI‐positive cells (Figure 3D and E) was significantly increased, while overexpression of TIPE2 reversed the effect of LPS/IL‐13 on bronchial epithelial cells. The above results indicated that overexpression of TIPE2 inhibited LPS/IL‐13‐induced pyroptosis of bronchial epithelial cells.

### 3.4 Overexpression of TIPE2 Inhibits Inflammation in LPS/IL‐13‐Treated Bronchial Epithelial Cells

**Figure 4:**
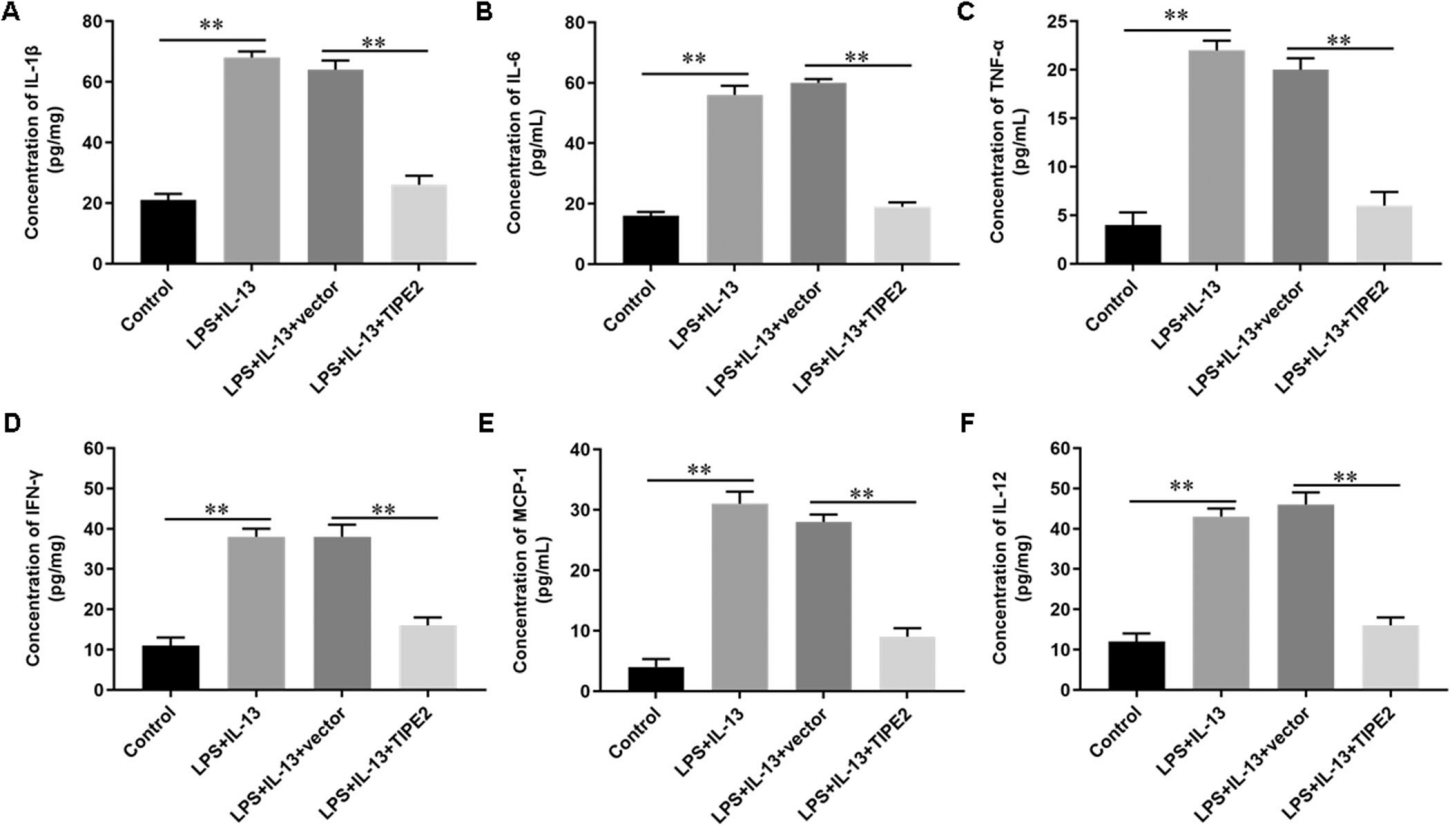
Overexpression of TIPE2 inhibits the expression of inflammatory factors in bronchial epithelial cells. Bronchial epithelial cells were incubated with 1 μg/mL LPS for 5 h, then incubated with 5.0 mM IL‐13 for 1 h, cultured at 37°C for 48 h. Next, cells were transfected with TIPE2 expression vector (pcDNA‐TIPE2) or pcDNA‐empty vector for 48 h and then cells were collected. A–F The concentration levels of IL‐1β (A), IL‐6 (B), TNF‐α (C), IFN‐γ (D), MCP‐1 (E) and IL‐12 (F) in the supernatant were detected by using ELISA. N = 5, *p < 0.05, **p < 0.01.

Pyroptosis is an inflammatory cell death that occurs with the release of a large number of pro‐inflammatory cytokines. In this study, we found that the concentration levels of IL‐1β, IL‐6, IL‐12, TNF‐α, IFN‐γ and MCP‐1 in the supernatant of bronchial epithelial cells were significantly increased after LPS/IL‐13 induction. Overexpression of TIPE2 significantly decreased the concentration levels of IL‐1β (Figure 4A), IL‐6 (Figure 4B), TNF‐α (Figure 4C), IFN‐γ (Figure 4D), MCP‐1 (Figure 4E) and IL‐12 (Figure 4F) in the supernatant of bronchial epithelial cells. These results suggested that overexpression of TIPE2 could significantly inhibit the release of pro‐inflammatory cytokines from bronchial epithelial cells induced by LPS/IL‐13. Therefore, we speculated that overexpression of TIPE2 might inhibit the pyroptosis of bronchial epithelial cells by inhibiting the inflammatory response.

### 3.5 Overexpression of TIPE2 Inhibits LPS/IL‐13‐Induced Activation of NLRP3 Inflammasome

**Figure 5:**
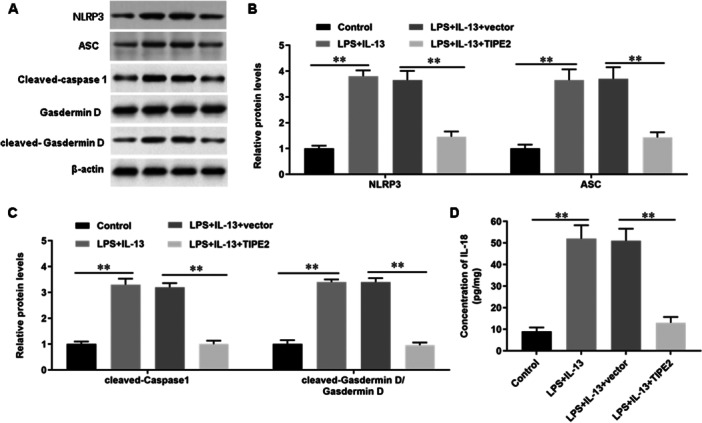
Overexpression of TIPE2 inhibits LPS/IL‐13‐induced activation of NLRP3 inflammasome. Bronchial epithelial cells were incubated with 1 μg/mL LPS for 5 h, then incubated with 5.0 mM IL‐13 for 1 h, cultured at 37°C for 48 h. Next, cells were transfected with TIPE2 expression vector (pcDNA‐TIPE2) or pcDNA‐empty vector for 48 h and then cells were collected. A–C Western blot analysis was used to detect the protein expression levels (A) of NLRP3, ASC (B), caspase‐1(p20) and cleaved‐Gasdermin D (C). (D) ELISA was used to detect the concentration of IL‐18. *N* = 5, *p < 0.05, **p < 0.01.

Then, to verify the effect of TIPE2 on LPS/IL‐13‐induced inflammation, TIPE2 was overexpressed in LPS/IL‐13‐incubated bronchial epithelial cells. The expression levels of NLRP3 inflammasome‐related proteins (Figure 5A) NLRP3, ASC, caspase‐1(p20) and cleaved‐Gasdermin D in bronchial epithelial cells were significantly increased under LPS/IL‐13 induction, while overexpression of TIPE2 inhibited the protein expression of NLRP3, ASC (Figure 5B), cleaved‐caspase‐1 and cleaved‐Gasdermin D (Figure 5C). In addition, we also found that overexpression of TIPE2 significantly inhibited the secretion of IL‐1β (Figure 4A) and IL‐18 (Figure 5D). These results suggested that overexpression of TIPE2 inhibited LPS/IL‐13‐induced activation of NLRP3 inflammasome.

### 3.6 Overexpression of TIPE2 Inhibits LPS/IL‐13‐Induced Oxidative Stress in Bronchial Epithelial Cells

**Figure 6:**
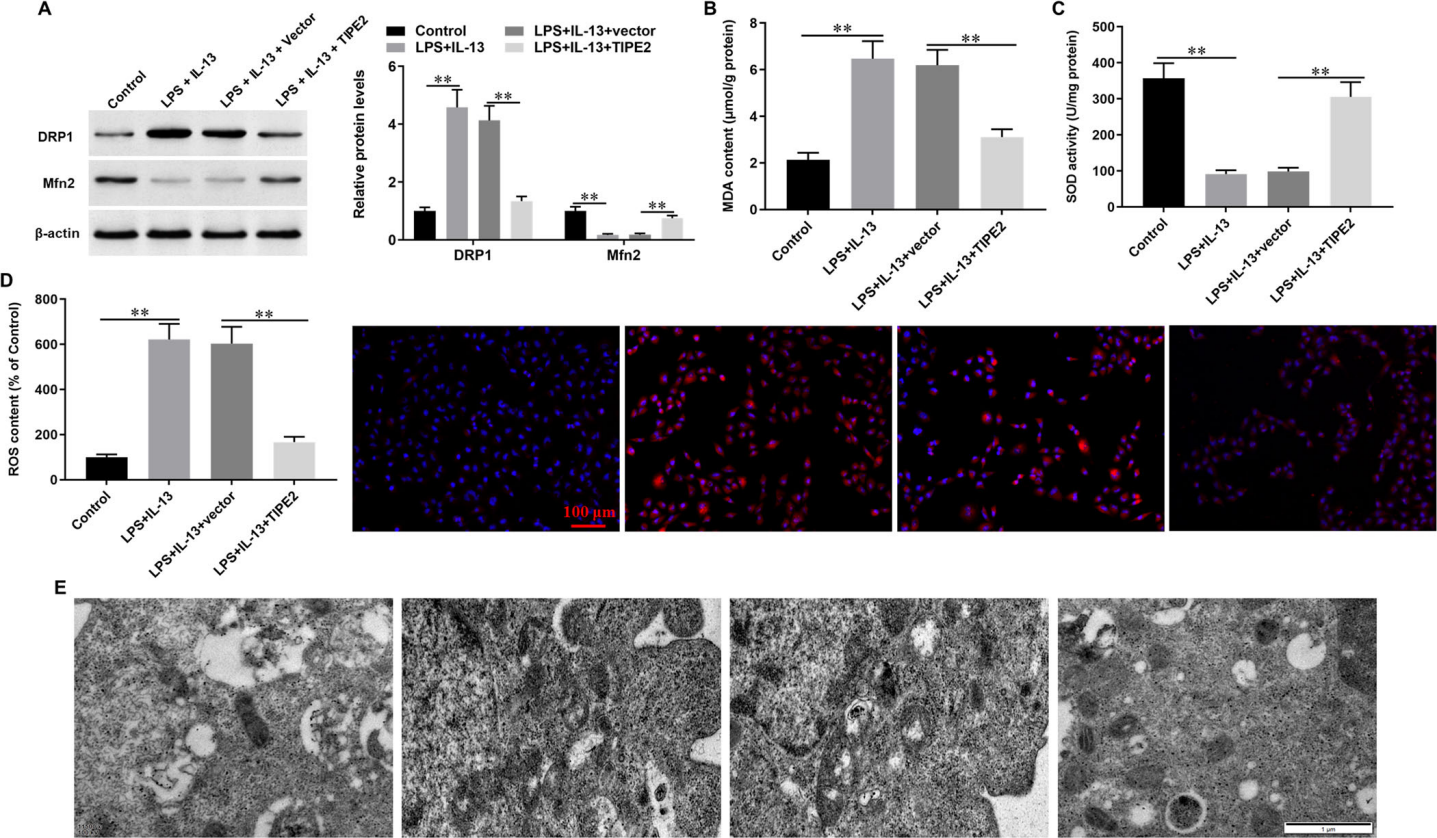
Overexpression of TIPE2 inhibits LPS/IL‐13‐induced oxidative stress. Bronchial epithelial cells were incubated with 1 μg/mL LPS for 5 h, then incubated with 5.0 mM IL‐13 for 1 h, cultured at 37°C for 48 h. Next, cells were transfected with TIPE2 expression vector (pcDNA‐TIPE2) or pcDNA‐empty vector for 48 h and then cells were collected. (A) Western blot analysis was used to detect the protein expression levels of DRP‐1 and Mfn2. (B, C) MDA production and SOD activity were detected with corresponding kits. (D) ROS fluorescence probe (DCFH‐DA) was used to evaluate the ROS content. (E) Transmission electron microscopy was used to observe the mitochondrial morphology. *N* = 3, *p < 0.05, **p < 0.01.

Then, the effect of TIPE2 on LPS/IL‐13‐induced oxidative stress was investigated. Our results showed that expression of mitochondrial fission marker protein dynamin‐related protein 1 (DRP‐1) was induced and mitochondrial fusion marker protein mitofusin 2 (MFN2) was suppressed by LPS/IL‐13 treatment, while overexpression of TIPE2 was able to decline the effect of LPS/IL‐13 on dysregulation of DRP‐1 and MFN2 (Figure 6A). Moreover, LPS/IL‐13 treatment promoted production of oxidative stress markers MDA (Figure 6B) and ROS (Figure 6D) and suppressed the activity of the antioxidant enzyme SOD (Figure 6C), which were significantly rescued by overexpression of TIPE2. Under transmission electron microscopy, we observed that LPS/IL‐13 treatment led to abnormal morphology of mitochondria, which was also rescued by overexpression of TIPE2 (Figure 6E).

### 3.7 TIPE2 Inhibits Activation of NLRP3 Inflammasome and C1qbp‐induced Oxidative Stress Through Interacting With the E3 Ubiquitin Ligase FBW1A

**Figure 7:**
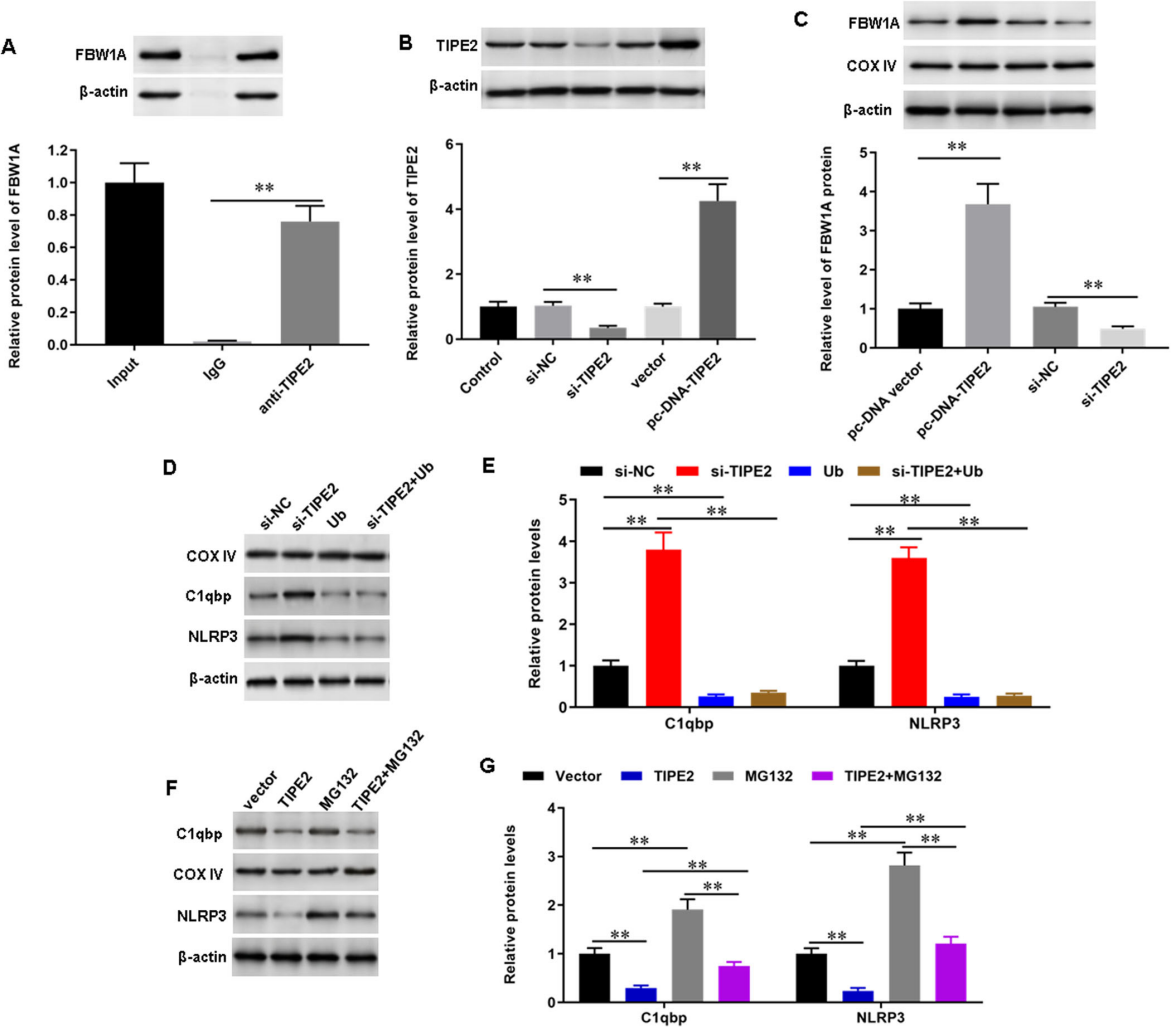
TIPE2 inhibits activation of NLRP3 inflammasome through interacting with FBW1A. (A) The interactions of TIPE2 and FBW1A were examined with co‐immunoprecipitation (Co‐IP). (B, C) si‐TIPE2 or pcDNA‐TIPE2 or their corresponding control was transfected into bronchial epithelial cells, the protein expression levels of TIPE2 (B) and FBW1A (C) were detected by using Western blot analysis. D,E Bronchial epithelial cells were treated with si‐TIPE2 and Ub individually or together, the protein expression levels of C1qbp and NLRP3 were detected by using Western blot analysis. (F, G) Bronchial epithelial cells were treated with pcDNA‐TIPE2 and MG132 individually or together, and the protein expression levels of C1qbp and NLRP3 were detected by using Western blot analysis. *N* = 3, *p < 0.05, **p < 0.01.

To explore the mechanism through which TIPE2 regulates bronchial epithelial cell pyroptosis, inflammation and oxidative stress, we searched potential target proteins with the online database Hitpredict (a resource of experimentally determined protein‐protein interactions, http://www.hitpredict.org/). To test this hypothesis, we conducted co‐immunoprecipitation (Co‐IP) experiments to examine the interaction of TIPE2 and E3 ubiquitin ligase FBW1A. The results showed that TIPE2 directly bound with FBW1A (Figure 7A), and TIPE2 positively regulated the expression of FBW1A protein (Figure 7B,C). As predicted in the online database Hitpredict and implied by the recent papers, NLRP3 and C1qbp were ubiquitinated substrates of FBW1A. To validate the involvement of FBW1A NLRP3 and C1qbp in TIPE2‐mediated pyroptosis, inflammation and oxidative stress, ubiquitination agonist Ub and inhibitor MG132 were applied to incubate TIPE2‐knocked down or TIPE2 overexpressed bronchial epithelial cells. The results showed that Ub diminished the effect of TIPE2 knockdown(Figure 7D–G). These data suggest that TIPE2 inhibits activation of NLRP3 inflammasome and C1qbp‐induced oxidative stress through interacting with the E3 ubiquitin ligase FBW1A.

### 3.8 Overexpression of TIPE2 Alleviates Lung Dysfunction in Asthmatic Mice

**Figure 8:**
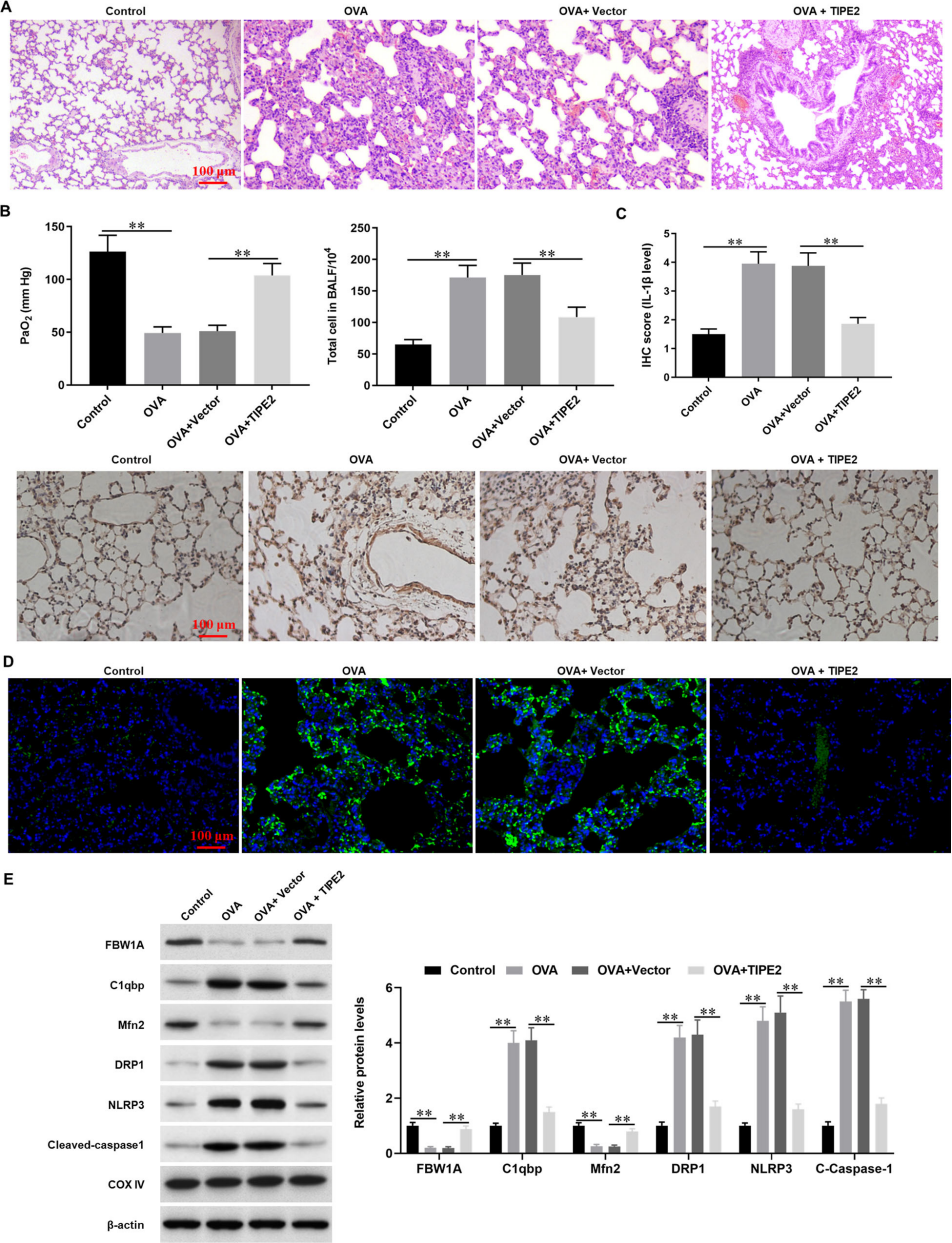
Overexpression of TIPE2 alleviates lung dysfunction in asthmatic mice. An asthmatic mouse model was established with OVA induction, and adenovirus‐mediated overexpression vector of TIPE2 (Ad‐TIPE2) or negative control (Vector) was applied to administrate the asthmatic mice. (A) HE staining was used to observe lung tissue morphology. (B) Cell counts in Bronchoalveolar Lavage Fluid (BALF) and PaO_2_ was used to assess the respiratory capacity of asthmatic mice. (C, D). Immunohistochemical and immunofluorescence assays were used to detect the expression of IL‐1β and TIPE2. (E) Western blot analysis was used to detect the expression levels of FBW1A, Mfn2, C1qbp, NLRP3, DRP1 and Cleaved‐caspase1 proteins in asthmatic mice. *N* = 4, *p < 0.05.

Finally, an asthmatic mouse model was established with OVA induction, and adenovirus‐mediated overexpression vector of TIPE2 (Ad‐TIPE2) was applied to administrate the asthmatic mice. HE staining, respiratory capacity assessment and IHC staining showed that TIPE2 overexpression improved cell counts in BALF, respiratory capacity (PaO_2_) and inflammation in asthmatic mice (Figure 8A–C). Immunofluorescence assay and Western blot analysis showed that overexpression of TIPE2 rescued the expression of FBW1A and Mfn2 and suppressed expression of C1qbp, NLRP3, DRP1 and Cleaved‐caspase1 in asthmatic mice (Figure 8D and E). These data demonstrated that overexpression of TIPE2 alleviates lung dysfunction in asthmatic mice.

## 4 Discussion

Tumor necrosis factor‐α‐induced protein‐8‐like 2 (TNFAIP8L2 or TIPE2) is one member of the TNFAIP8 family. Current evidence suggests that TIPE2 is a negative regulator of innate and adaptive immune responses and is preferentially expressed by immune cells. TIPE2 may play important roles during the pathogenesis of autoimmune and allergy diseases [18]. Glucocorticoids are known to induce cell apoptosis and affect many human physiological systems, including nervous, skeletal, muscular, endocrine, circulatory, and the immune system, and a recent study demonstrated that TIPE2 facilitated glucocorticoid‐mediated cell apoptosis in mouse thymocytes [19]. Another study showed that TIPE2‐deficient macrophages had enhanced phagocytic and bactericidal activities, in which case TIPE2‐knockout mice are resistant to bacterial infection and acquire higher survival ability [20]. TIPE2 was reported to ameliorate lipopolysaccharide‐induced apoptosis and inflammation and suppress the activation of NF‐κB and JNK in acute lung injury [21]. Moreover, several recent reports have indicated that TIPE2 functioned as a suppressor in asthma by regulating inflammation of multiple cell types, such as airway smooth muscle cells, macrophages, and Tregs [22, 23, 24]. However, the exact role and underlying mechanism of TIPE2 in the process of bronchial epithelial cell dysfunction remain largely unknown. In this study, we screened TIPE2 as a key regulator in bronchial epithelial cells in asthma. Its expression was decreased by LPS/IL‐13 incubation in a time‐dependent manner, and overexpression of TIPE2 suppressed bronchial epithelial cell pyroptosis, inflammation, and oxidative stress by interacting with the E3 ubiquitin ligase FBW1A.

FBW1A, also beta‐Trcp, is an important member of the Skp1/cullin/F‐box proteins (SCF) class of E3 ubiquitin ligases, which was frequently recruited by Akt‐mTOR complexes during cellular stresses, including autophagy, endoplasmic reticulum stress, and oxidative stress in cancerous and nonmalignant tissues [25, 26, 27]. As the largest family of cullin‐RING ligases, SCFs play an important role in pathophysiology of various tissues by regulating innate immunity and inflammation. SCFs have been demonstrated to be dysregulated in inflammatory lung disease and display a protective role in lung endothelial cells [28, 29]. As a key member of F‐box proteins, FBW1A exhibited suppressive roles in regulating the expression of pro‐inflammatory transcription factors such as TGF‐beta and NF‐kappa B [30]. However, the role of FBW1A in asthma is not clear. In this study, we reported that upregulation of the E3 ubiquitin ligase FBW1A, induced by overexpression of TIPE2, could significantly suppressed expression of NLRP3 and NLRP3‐mediated pyroptosis and inflammation in vitro and vivo. Additionally, FBW1A interacted with TIPE2, and it functioned as a negative regulator in bronchial epithelial cell pyroptosis, inflammation, and oxidative stress, may through degrading the NLRP3 and C1qbp proteins. To our knowledge, this is the first report that reveals the exact role of FBW1A in asthma.

C1qbp is a famous mitochondrial protein that plays a critical role in mitochondrial oxidation [31]. In cancer tissues, C1qbp was mostly reported as a proto‐oncogene to enhance cell survival, migration, invasion, and therapeutic resistance [32, 33, 34]. In inflammatory diseases, C1qbp usually contributed to excessive production of ROS and oxidative stress [35, 36]. However, in respiratory inflammatory diseases, the role of C1qbp was largely unknown. Recent reports implied that C1qbp was closely associated with AKT/mTOR/SCF complex and might be regulated by SCF [34]. In this study, we found that C1qbp was involved in LPS/IL‐13‐induced oxidative stress of bronchial epithelial cells. Upregulation of the E3 ubiquitin ligase FBW1A, induced by overexpression of TIPE2 could significantly suppressed expression of C1qbp and NLRP3‐mediated oxidative stress in vitro and vivo.

In summary, TIPE2 protects bronchial epithelial cells from NLRP3‐induced pyroptosis and C1qbp‐induced mitochondrial damage and oxidative stress by interacting with the E3 ubiquitin ligase FBW1A in asthmatic mice.

## Author Contributions

**Wei Li:** funding acquisition, conceptualization, writing – original draft. **Yonghong Zhang:** methodology. **Yuanyuan Wu:** methodology. **Dan Wang:** investigation. **Juan Ge:** formal analysis. **Hongyan Zhao:** investigation. **Yun Liu:** writing – review and editing, supervision.

## Ethics Statement

This study was approved by the Ethics Committee of Xi'an Jiaotong University.

## Conflicts of Interest

The authors declare no conflicts of interest.

## Data Availability

The data that support the findings of this study are available from the corresponding author upon reasonable request.
